# Study protocol for a stepped-wedge randomized cookstove intervention in rural Honduras: household air pollution and cardiometabolic health

**DOI:** 10.1186/s12889-019-7214-2

**Published:** 2019-07-08

**Authors:** Bonnie N. Young, Jennifer L. Peel, Megan L. Benka-Coker, Sarah Rajkumar, Ethan S. Walker, Robert D. Brook, Tracy L. Nelson, John Volckens, Christian L’Orange, Nicholas Good, Casey Quinn, Joshua P. Keller, Zachary D. Weller, Sebastian Africano, Anibal B. Osorto Pinel, Maggie L. Clark

**Affiliations:** 1 Department of Environmental and Radiological Health Sciences, Colorado State University, Fort Collins, CO 80523-1681 USA; 2 Department of Health Sciences, Gettysburg College, Gettysburg, PA USA; 3 Division of Cardiovascular Medicine, University of Michigan Medical School, Ann Arbor, MI USA; 4 Department of Health and Exercise Science, Colorado State University, Fort Collins, CO USA; 5 Department of Mechanical Engineering, Colorado State University, Fort Collins, CO USA; 6 Department of Statistics, Colorado State University, Fort Collins, CO USA; 7 Trees, Water & People, Fort Collins, CO USA; 8 Asociación Hondureña para el Desarrollo, Tegucigalpa, Honduras

**Keywords:** Biomass fuel, Household air pollution, Particulate matter, Personal exposure, Cardiovascular health, Metabolic health, C-reactive protein, Blood pressure, Hemoglobin A1c, Randomized controlled trial

## Abstract

**Background:**

Growing evidence links household air pollution exposure from biomass-burning cookstoves to cardiometabolic disease risk. Few randomized controlled interventions of cookstoves (biomass or otherwise) have quantitatively characterized changes in exposure and indicators of cardiometabolic health, a growing and understudied burden in low- and middle-income countries (LMICs). Ideally, the solution is to transition households to clean cooking, such as with electric or liquefied petroleum gas stoves; however, those unable to afford or to access these options will continue to burn biomass for the foreseeable future. Wood-burning cookstove designs such as the *Justa* (incorporating an engineered combustion zone and chimney) have the potential to substantially reduce air pollution exposures. Previous cookstove intervention studies have been limited by stove types that did not substantially reduce exposures and/or by low cookstove adoption and sustained use, and few studies have incorporated community-engaged approaches to enhance the intervention.

**Methods/design:**

We conducted an individual-level, stepped-wedge randomized controlled trial with the *Justa* cookstove intervention in rural Honduras. We enrolled 230 female primary cooks who were not pregnant, non-smoking, aged 24–59 years old, and used traditional wood-burning cookstoves at baseline. A community advisory board guided survey development and communication with participants, including recruitment and retention strategies. Over a 3-year study period, participants completed 6 study visits approximately 6 months apart. Half of the women received the *Justa* after visit 2 and half after visit 4. At each visit, we measured 24-h gravimetric personal and kitchen fine particulate matter (PM_2.5_) concentrations, qualitative and quantitative cookstove use and adoption metrics, and indicators of cardiometabolic health. The primary health endpoints were blood pressure, C-reactive protein, and glycated hemoglobin. Overall study goals are to explore barriers and enablers of new cookstove adoption and sustained use, compare health endpoints by assigned cookstove type, and explore the exposure-response associations between PM_2.5_ and indicators of cardiometabolic health.

**Discussion:**

This trial, utilizing an economically feasible, community-vetted cookstove and evaluating endpoints relevant for the major causes of morbidity and mortality in LMICs, will provide critical information for household air pollution stakeholders globally.

**Trial registration:**

ClinicalTrials.gov Identifier NCT02658383, posted January 18, 2016, field work completed May 2018. Official title, “Community-Based Participatory Research: A Tool to Advance Cookstove Interventions.” Principal Investigator Maggie L. Clark, Ph.D. Last update posted July 12, 2018.

**Electronic supplementary material:**

The online version of this article (10.1186/s12889-019-7214-2) contains supplementary material, which is available to authorized users.

## Background

### Background and study aims

Household air pollution is a major global health problem, as nearly 3 billion people rely on the burning of solid fuels (e.g., wood, charcoal) for cooking [1]. The combustion of biomass fuel in inefficient and poorly vented cookstoves creates a complex mixture of pollutants, which includes particulate matter (PM), carbon monoxide, volatile organic compounds, polycyclic aromatic hydrocarbons, and nitrogen oxides [2, 3]. Households cooking with biomass fuels experience PM_2.5_ levels (fine particles < 2.5 μm in aerodynamic diameter) 10 to 50 times higher than the World Health Organization’s current guideline of 25 μg/m^3^ for a 24-h mean [4, 5]. Household air pollution was estimated to cause 60 million disability-adjusted life years and 1.6 million premature deaths globally in 2017 [6].

Despite being a leading risk factor for morbidity and mortality worldwide, a complete understanding of the burden from household air pollution is unknown, as only a limited number of health outcomes are included in global burden estimates. Estimates of cardiovascular-related outcomes are almost entirely extrapolated from exposure-response effects associated with other sources of combustion-related pollution, such as active smoking, secondhand smoke, and ambient air pollution [6]. Growing evidence links household air pollution with increased risk for cardiovascular diseases (CVDs) through potential inflammatory and oxidative stress pathways [7–13]. Exposure to household air pollution is also gaining recognition for its potential impact on type 2 diabetes, with proposed mechanisms linking air pollution and metabolic dysfunction through chronic inflammation [14]. Further investigation is needed to more accurately characterize the burden of disease from household air pollution, particularly its impact on cardiometabolic diseases, which are among the leading causes of global morbidity and mortality [6].

Ideally, the solution to elevated household air pollution exposure is to transition households to clean cooking, such as with electric or liquefied petroleum gas stoves. However, those unable to afford or access these options will continue to burn biomass for the foreseeable future. Solid-fuel cookstoves designed with engineered combustion chambers and chimneys have the potential to reduce emissions and evidence suggests that the introduction of these stoves is capable of improving health. For example, the first randomized controlled trial evaluating the health impact of a chimney stove demonstrated a reduction in severe childhood pneumonia in Guatemala [15]. The intervention in Guatemala also resulted in clinically meaningful reductions in blood pressure levels among women [7]. Despite the potential to reduce household air pollution and improve health, the majority of cookstove programs (i.e., programs disseminating clean stoves and cookstoves designed to burn solid fuels more efficiently) around the world have not demonstrated consistent evidence for health-related benefits [16]. Achievable exposure reductions depend largely on a wide range of factors influencing adoption and sustained use of a newly introduced cookstove, including cultural, financial, geographical, familial, and individual [17, 18]. Low acceptance of new cookstoves, resistance to change cooking behaviors, inability of new stoves to meet the needs of the family, and continued use of traditional cookstove technologies have been observed across numerous cookstove interventions [17, 19–21]. While difficult to achieve in reality, near-complete displacement of traditional cookstoves is considered necessary to maximize health benefits [22, 23].

In our 2015 cross-sectional study among women in the same rural Honduran target population, we observed that 24-h mean kitchen and personal PM_2.5_ concentrations were 61 and 47% lower, respectively, among *Justa* cookstove users (i.e., engineered combustion zone and chimney stoves) compared to traditional cookstove users [24]. According to a simulated liquified petroleum gas stove intervention that assumed PM_2.5_ concentrations were reduced to 70 μg/m^3^, our 24-h *Justa* cookstove PM_2.5_ concentrations (average [median, IQR] personal PM_2.5_ concentration was 66 μg/m^3^ [53, 39 to 81 μg/m^3^]) [24] were within a range that should confer health benefits, particularly for systolic blood pressure [25]. Our cross-sectional measures of household air pollution were associated with elevated blood pressure (e.g., adjusted mean systolic blood pressure was 2.5 mmHg higher (95% confidence interval [CI], 0.7–4.3) per unit increase in natural log-transformed kitchen PM_2.5_), with suggestive evidence of associations when stove type (i.e., *Justa* compared to traditional cookstove use) was the exposure of interest [26]. We further observed cross-sectional effects of household air pollution on increased prevalence of metabolic syndrome (prevalence ratio per interquartile range increase in kitchen PM_2.5,_ 1.16 [95% CI, 1.0–1.3]) [24] and prediabetes/diabetes (prevalence ratio per interquartile range increase in kitchen PM_2.5_, 1.49 [95% CI, 1.1–2.0]); results were generally in the hypothesized direction for glycated hemoglobin (HbA1c) [27]. However, no evidence of association was observed with these outcomes when stove type was the exposure of interest [27]. Finally, there was evidence for a greater prevalence of self-reported symptoms (i.e., an indicator of quality of life) among traditional cookstove users compared to *Justa* users [28].

Despite the observed effects of household air pollution on cardiometabolic disease indicators from our formative study, residual confounding and lack of temporality are major limitations to cross-sectional designs, calling for the need for a randomized study with repeated measures of exposure and health outcomes. Although evidence suggests improvements in health following reductions in ambient air pollution exposures, much less is known about whether or not efforts to reduce exposures to emissions from household solid fuel combustion will result in health benefits, and if certain subgroups of the population are more likely to see improvements [29]. Furthermore, a better understanding is also needed on the barriers surrounding cookstove adoption and sustained use [18]. We propose that sustained use of a carefully selected, culturally appropriate, wood-burning cookstove with an engineered combustion chamber and chimney (the *Justa* cookstove) will result in lower air pollution exposures and better health status compared to the use of the traditional cookstove. Within a community-engaged framework among rural Honduran women, this cookstove intervention addresses the following aims through an individual-level, stepped-wedge randomized controlled trial:

### Aim 1: in an intent-to-treat framework, evaluate the impact of the *Justa* cookstove intervention on indicators of cardiometabolic disease risk

Among 230 female primary cooks, we will evaluate 3 primary health endpoints (blood pressure, C-reactive protein [CRP], and HbA1c) among those randomly assigned to using traditional and *Justa* cookstoves during a 3-year project with 6 study visits, spaced approximately 6 months apart. Secondary health endpoints include indicators of other cardiometabolic- and respiratory-related outcomes, specifically metabolomics and other biomarkers of systemic injury and inflammation from dried blood spots, augmentation index, central pulse pressure, blood lipids, self-reported health symptoms, fractional exhaled nitric oxide, and telomere length from buccal cells. We will evaluate the potential for effect modification by age, blood pressure, diabetes, body mass index (BMI), waist circumference, and metabolic syndrome. *We hypothesize that use of the Justa cookstove (intervention) will result in improved indicators of non-communicable disease risk as compared to use of the traditional cookstove among Honduran women.*

### Aim 2: in an exposure-response framework, characterize the associations between household air pollution measurements and indicators of cardiometabolic disease risk

To explore the link between exposure to household air pollution and indicators of cardiometabolic disease risk, we will utilize a longitudinal design with 6 repeated measures of 24-h kitchen and personal PM_2.5_ and measures of primary and secondary outcomes (indicators of cardiometabolic- and respiratory-related outcomes as listed above in Aim 1) collected every 6 months over a 3-year study period among the 230 study participants. We will evaluate the potential for effect modification by age, blood pressure, diabetes, BMI, waist circumference, and metabolic syndrome. *We hypothesize a positive exposure-response relationship between PM*_*2.5*_ *and indicators of cardiometabolic- and respiratory-related outcomes. We expect the characterization of the exposure-response to be transferable to other settings/stove types; thus providing the potential to inform decisions of acceptable levels of household air pollution regardless of cooking technology.*

## Methods

### Study setting

This research builds on existing partnerships between Colorado State University (CSU) and Trees, Water & People (TWP) in Fort Collins, Colorado, USA, and the Honduran Association for Development (Asociación Hondureña para el Desarrollo, AHDESA) in Tegucigalpa, Honduras. The study area included 10 rural communities near the town of La Esperanza in the Department of Intibucá, Honduras. Intibucá is a mountainous region with elevations ranging from approximately 1700 to 2200 m. The study area has an agricultural-based economy, farming potatoes, beans, coffee, and other fruits and vegetables. Households in the region were dependent on biomass fuel (wood) for cooking. Primary and secondary stove types varied from 3-stone fires to modified traditional cookstoves with griddles and chimneys. Most kitchens were enclosed and located in the main house or as a separate building near the main house, and most secondary stoves were located outside.

### Formative research and cookstove selection

**Fig. 1:**
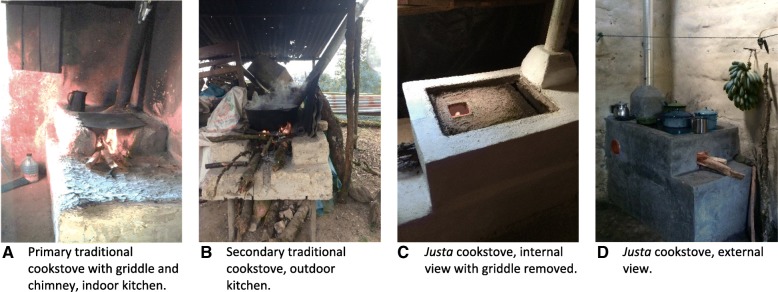
Examples of a primary traditional cookstove (**a**) and secondary traditional cookstove (**b**), and internal and external views of *Justa* cookstoves (**c**, **d**)

In fall 2014, we prepared for the trial by assessing the study population with a convenience sample of over 500 in-person surveys on sociodemographic characteristics, cookstove types, preferences of cookstove models, obstacles to new cookstove adoption, cooking behaviors, and cookstove experts’ perceptions of successes and failures of previous interventions (NIH ES022269). These findings, along with input from our community partners at TWP and AHDESA, supported the selection of the wood-burning *Justa* cookstove for the intervention. The *Justa* cookstove was well-accepted, culturally appropriate, locally sourced, and functioned well when maintained correctly. It was designed specifically for Honduran homes with an insulated rocket-elbow ceramic combustion chamber, chimney, metal griddle, and side soot compartment [30]. The griddle can hold 2 to 3 pots at once, and is also used for making tortillas. Examples of traditional and *Justa* cookstoves are shown in Fig. 1.

As the next step in our formative research, we conducted a cross-sectional study in spring 2015 among 150 women from the same study population (NIH ES022810). Half of the women were using a *Justa* cookstove received from other projects, and the other half cooked on traditional cookstoves. This study allowed us to test our equipment in the field and refine our data collection procedures. As mentioned above, we observed evidence of associations between exposure to household air pollution and blood pressure [26], prediabetes/diabetes [27], and metabolic syndrome [24], as well as self-reported symptoms among traditional cookstove users compared to *Justa* users [28].

### Trial design

We utilized an individual-level, stepped-wedge randomized controlled trial design, with 230 women using traditional wood-burning cookstoves at baseline. Women were randomly assigned into study arms 1 and 2 by blindly drawing a number from a bag at a community meeting. Six repeated measures occurred over the 3-year study period from August 2015 through May 2018 (Table 1). Study arm 1 (*n* = 115) received the *Justa* cookstove intervention after visit 2, and arm 2 (*n* = 115) received the intervention after visit 4 (Table 1). A unique benefit of the stepped-wedge design is that each study arm provides before and after observations, and each arm switches from ‘control’ status to ‘intervention’ status over the course of the study, but not at the same time [31].

**Table 1:** Study scheme for timing and visits of interventions, Honduras, August 2015 – May 2018

Stepped-wedge randomized controlled trial		
Timing of study visits for data collection	Arm 1 (*n*=115) Stove type	Arm 2 (*n*=115) Stove Type
Visit 1: Aug – Dec 2015	Traditional	Traditional
Visit 2: Jan – May 2016	Traditional	Traditional
	Arm 1 receives intervention	
Visit 3: Sep – Dec 2016	*Justa*	Traditional
Visit 4: Feb – May 2017	*Justa*	Traditional
		Arm 2 receives intervention
Visit 5: Sep – Dec 2017	*Justa*	*Justa*
Visit 6: Feb – May 2018	*Justa*	*Justa*

### Recruitment and retention

Communities were selected with guidance from the community advisory board based on traditional cookstove use among households, accessibility from the field house in La Esperanza (within an hour drive), and permission from local leaders to conduct the study within their community. Eligibility to participate included the following criteria: female, 24–59 years of age, primary cook of the household, not pregnant at time of recruitment, non-smoking, not exposed to secondhand smoke, used only a biomass fuel traditional cookstove for cooking. Community meetings were held to introduce the research team and objectives and to obtain a list of people’s names who were interested in enrolling in the study. Women not in attendance at the meeting could still be enrolled if their name was written by a friend or family member, or if they later decided they would like to be screened for eligibility. After the initial study visit, a woman was skipped for data collection if she was pregnant or not home after 2 attempts and revisited for the following study visits.

To motivate women to continue participating for all 6 visits, we offered an incentive of a bag of food items at each visit worth $5 USD. We also offered a one-time secondary incentive of similar perceived value to the intervention cookstove to arm 2 at the time when arm 1 received the *Justa* cookstove, and then to arm 1 at the time when arm 2 received the *Justa* cookstove. The community advisory board helped select secondary incentive options of a radio, kitchen utensils, or a basket of specialty food items, with the criteria that the gift would not influence exposure.

### Implementation and training

Primary materials for the *Justa* intervention cookstoves included a chimney, griddle, combustion chamber and soot compartment, manufactured by AHDESA in Tegucigalpa and donated by the Fondo Centroamericano para el Acceso a la Energía y Reducción de la Pobreza (FOCAEP). Additional materials for stove construction included cement blocks, which were acquired locally in La Esperanza, and households were asked to contribute a table to hold the cookstove (usually a large, immobile, self-constructed adobe base), sifted wood ash, a cardboard box, nails, wooden boards and mud.

Community meetings were held during the first study visit in every village to review the cookstove construction process and discuss the required materials that each family would procure. Participants were required to destroy their traditional cookstove before the new *Justa* cookstove was built, although they could keep their previous base (i.e., table) if it fit the necessary dimensions for the new *Justa* cookstove. A second community meeting was held among study arm 2 participants prior to their *Justa* construction to review the cookstove construction process and discuss the materials. Cookstoves were constructed by AHDESA technicians and the study coordinators.

A few weeks before the first *Justa* cookstoves were constructed, each community received 2 new *Justa* cookstoves for their local elementary schools, donated by the United States Agency for International Development. All participants were invited to observe and ask questions during the construction and testing of the new cookstoves.

Personal training on cookstove use and maintenance occurred for each woman during the *Justa* cookstove construction in her home. The training was identical for all participants, as trainers followed a checklist and review. The training covered the identification of cookstove parts, choice of fuel, fuel size, cleaning, and the maintenance schedule. The women were given a water-resistant poster that provided a summary of the stove maintenance steps and a telephone contact for additional help (Additional file 1: Figure S1). The posters were hung in the kitchens near the new cookstoves. These educational materials and training steps were developed through the guidance of the community advisory board, TWP, and AHDESA. Community leaders for each village served as points of contact for study participants and were informed about the project’s progress at monthly meetings with the research team.

At every study visit following the intervention, the condition and maintenance of the *Justa* cookstove were assessed through women’s self-report and direct researcher observations. The survey questions were identical to the maintenance steps outlined in the educational poster. If certain steps were not being properly followed, the researcher would re-explain the steps and emphasize the reason for the maintenance.

### Study visits

A typical visit involved arriving at the first house for set-up around 7:30 am, greeting the woman, obtaining verbal informed consent by our study coordinator, installing all exposure monitors, and conducting health and kitchen questionnaires. This day 1 set-up usually lasted around 20–25 min. The following day (at least 24 h later), the research team would return to the woman’s house, remove all exposure equipment, complete the health questionnaire and stove maintenance questions, take all health measures, give the woman her incentive, and explain her health results to her (i.e., blood pressure, BMI, HbA1c, cholesterol, and triglycerides). We explained health results to the women using categories of normal/abnormal based on established cut points; however, women were told that the researchers were not medical professionals and could not make diagnoses based on health results, and if she had any worries or questions, she should visit her health center to speak with a healthcare provider (Additional file 2: Figure S2). This day 2 visit usually lasted between 40 and 50 min.

All data collection was completed by 12 noon each day. Study visits took place Monday through Saturday. No samples were collected on Sundays due to the potential to capture atypical cooking behaviors.

### Exposure assessment

**Fig. 2:**
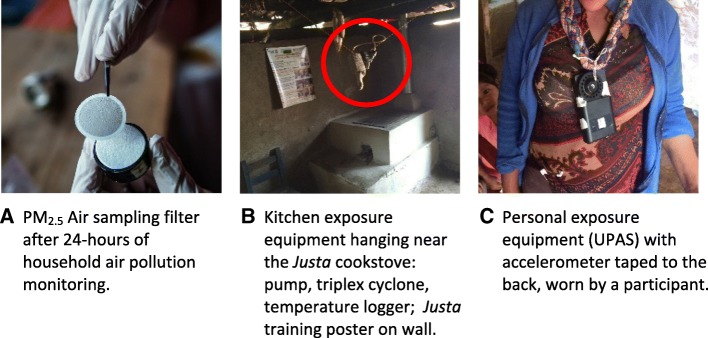
Examples of exposure collections: post-sampling filter (**a**), kitchen exposure monitors near cookstove (**b**), and personal UPAS monitor (**c**). Photo credits: Joanna B. Pinneo (**a**)

Table 2 summarizes all exposure assessments. Our main exposures of interest were assigned cookstove type categories (*Justa* cookstove versus traditional cookstove; Fig. 1), and 24-h gravimetric personal and kitchen household air pollution concentrations (PM_2.5_). Kitchen air pollution monitors were placed within a range of 76–127 cm above the front edge of the cookstove, slightly above the woman’s breathing zone when standing at the stove to avoid interference with her cooking tasks, and away from the direct plume of smoke and windows and doorways (Fig. 2). Kitchen temperature and relative humidity data were measured during each visit and the monitors (Lascar electronics data logger, Erie, PA, USA) were collocated with the kitchen exposure equipment. Personal air pollution monitors were clipped onto a small bag or cloth necklace near the woman’s breathing zone and worn for 24 h, with instructions to only remove the bag/necklace to sleep and bathe and to keep the bag/necklace near the bed while sleeping (Fig. 2). Field blanks were collected once a week. An accelerometer that logged 3-axis movement data was collocated with the personal exposure equipment to estimate compliance of wearing the monitors (X16-1D Accelerometer, Gulf Coast Data Concepts, Waveland, MS, USA).

**Table 2:** Summary of health, exposure, and other participant and household measurements

Measurement	Description	Source	Groups Measured	Timing^a^
Primary health endpoints	Blood pressure (brachial systolic and diastolic) C-reactive protein (systemic inflammation) Glycated hemoglobin (HbA1c)	SphygmoCor XCEL monitor Dried blood spots Finger-stick point-of-care instrument	All participants All participants All participants	All 6 visits, day 2 All 6 visits, day 2 All 6 visits, day 2
Secondary health endpoints	Other biomarkers of systemic injury and inflammation Metabolomics Augmentation index and central pulse pressure Blood lipids (LDL, HDL, total cholesterol, triglycerides) Self-reported health symptoms Fractional exhaled nitric oxide (airway inflammation) Telomere length from buccal cells	Dried blood spots Dried blood spots SphygmoCor XCEL Finger-stick point-of-care instrument Questionnaires NIOX Vero and Aero monitors Buccal scrapes	All participants All participants All participants All participants All participants Sub-set of participants Sub-set of participants	All 6 visits, day 2 Visit 1 All 6 visits, day 2 All 6 visits, day 2 All 6 visits, day 2 All 6 visits, day 2 Visits 1-4, day 2
Exposure assessments	Assigned stove type	Randomization	All participants	See Table 1 scheme
	Kitchen			
	PM_2.5_ Black carbon Ultrafine PM Real time and gravimetric PM_2.5_ Carbon monoxide	Triplex cyclone and AirChek pump PM_2.5_ Filters and Sootscan Discmini Personal Data Ram Dräger Pac 7000	All kitchens All kitchens Sub-set of kitchens Sub-set of kitchens All kitchens	All 6 visits, 24-hours All 6 visits, 24-hours Visits 1-4, 24-hours Visits 1-4, 24-hours Visits 1-3, 24-hours
	Personal			
	PM_2.5_ Black carbon Compliance with wearing monitors Carbon monoxide	Triplex cyclone and AirChek pump, UPAS PM_2.5_ Filters and Sootscan Accelerometer Dräger Pac 7000	All participants All participants All participants All participants	All 6 visits, 24-hours All 6 visits, 24-hours All 6 visits, 24-hours Visits 1-3, 24-hours
Other sources of household air pollution	Neighbors, traffic, trash burning, kerosene use Smoking status (cigarettes and puro) Secondhand smoke exposure	Questionnaires Questionnaires Screening	All participants All participants All participants	All 6 visits, day 1 Screening and all 6 visits, day 2 Screening
Stove use	Self-reported use of all cookstoves, behaviors Electronic monitoring of cookstove temperature *Justa* cookstove maintenance, changes, preferences Time cooking separately for animals	Questionnaires Stove Use Monitors (SUMs) Questionnaires Questionnaires	All participants All cookstoves *Justa* recipients All participants	All 6 visits, day 2 All 6 visits, 24-hours Visits 3-6, day 2 All 6 visits, day 2
Household and kitchen characteristics	Elevation and GPS coordinates Kitchen temperature and humidity Kitchen ventilation/openings Kitchen location, size, and housing materials	Maps.me on smart phones EasyLog monitor Questionnaires Questionnaires	All households All kitchens All kitchens All kitchens	Visit 1 (repeated if new house) All 6 visits, 24-hours Visit 1 (repeated if new house) Visit 1 (repeated if new house)
Socioeconomic and demographics	Income sources Material items, electricity, education, beds, age Household size, age of youngest child	Questionnaires Questionnaires Questionnaires	All participants All participants All participants	All 6 visits, day 1 Visit 1, day 1 All 6 visits, day 1
Other health-related measures	Illnesses, previous diagnoses Medication and vitamin use 24-hour dietary recall and diversity Weekly physical activity Body mass index (BMI), waist and hip circumference Pregnancy	Questionnaires Questionnaires Questionnaires Questionnaires Anthropometric measures Screening and Questionnaires	All participants All participants All participants All participants All participants All participants	All 6 visits, day 2 All 6 visits, day 2 All 6 visits, day 2 All 6 visits, day 2 All 6 visits, day 2 Screening and all 6 visits, day 1

^a^Women received six repeated measures over the 3-year study period, with approximately six months between visits. Each study visit included two consecutive days of data collection: day 1 set-up of all exposure monitors, and day 2 take-down of monitors and collection of health measures. Further details on timing and collection procedures can be found in the “Study visits” section

Twenty-four hour kitchen and personal PM_2.5_ samples were collected on 37 mm filters (Fiberfilm, Pall Corporation, NY, USA and Teflo filters, VWR, Radnor, PA, USA). PM_2.5_ was sampled by drawing air through a size-selective cyclone inlet (Triplex, BGI, Inc., NJ, USA) using a pump (AirChek XR5000, SKC Inc., PA, USA) calibrated to 1.5 liter (L) per minute (DryCal Lite, Mesa Labs, NJ, USA) prior to each sample. Due to product availability, we switched from Pallflex fiberfilm filters to Teflo filters prior to exposure monitoring for the fifth study visit. Our filter comparison study demonstrated excellent agreement and correlation between paired samples collected in Honduran kitchens (Pearson correlation coefficient, 0.96; *n* = 16 paired observations).

We switched to a different personal exposure monitor starting at the fifth study visit for all participants, called the Ultrasonic Personal Aerosol Sampler (UPAS, Access Sensor Technologies, Fort Collins, CO, USA) (Fig. 2). The UPAS included a miniature piezoelectric pump to draw in air at 1.0 L/minute, with a customized cyclone that captured PM_2.5_ sample on the enclosed filter. It is more compact, lighter (198 g), and less noisy than the original setup [32]. To ensure consistency between the previously used filter sampling system and the UPAS, we conducted a field evaluation in spring 2017 [33]. We observed strong agreement and correlation (Spearman coefficient 0.91) between 43 paired measures [33].

Filters from kitchen and personal PM_2.5_ sampling were stored at − 20 °C prior to transport from Honduras to CSU for post-sampling analysis. At CSU, filters were stored at − 80 °C, equilibrated to weighing-room conditions over at least 24 h prior to analysis, and analyzed gravimetrically to determine PM_2.5_ mass (Mettler Toledo MX5 Microbalance, Mettler Toledo, Columbus, OH, USA). Mass measurements were made in duplicate (or triplicate if the first 2 values differed by more than 5 μg) and averaged. For each sample, PM_2.5_ mass was calculated as the difference between the post- and pre-sampling measurements. The PM_2.5_ limit of detection (LOD) for each phase was estimated by adding the mean mass of the field blanks to 3 times the standard deviation of the field blank masses [34]. Samples that were below the LOD were substituted with the LOD/$\sqrt{2}$. Samples were blank-corrected by subtracting the mean blank mass for the phase. Final 24-h PM_2.5_ concentrations were estimated by dividing the blank-corrected filter mass by the volume of the air sampled through the pump over the measurement period. Pump performance was considered adequate if the calibration flow rate had less than 10% difference between pre- and post-sampling measurements.

Samples were analyzed for black carbon using the same 37 mm Pallfex and Teflo filters as the PM_2.5_ samples. Black carbon concentrations were estimated based on the change in optical transmission of 880 nm light through the filters [35] before and after sampling (Transmissometer model OT-21, Magee Scientific, Berkeley, CA, USA). Pre-sample transmission was measured for study visits 2–6, and estimated for study visit 1. Full methods for black carbon estimation, including measures of attenuation, reference values for transmittance, LOD calculations, and final calculations have been previously described in detail [26].

Time-resolved (1 Hz) particle number concentration (PNC) was measured with a diffusion disc classifier (Discmini, Matter Aerosol, Wohlen, Switzerland), placed in the kitchen for 24-h. Due to having only one PNC instrument, the Discmini was deployed on Mondays, Wednesdays, and Fridays in a subset of 20–40 women during visits 1 to 4. The Discmini was placed within 102 to 178 cm from the front edge of the stove and not directly in front of a window or door. Real-time and gravimetric PM_2.5_ (in addition to the primary PM_2.5_ measurements described above) were measured with the Personal Data Ram (PDR 1200, Thermo Electron Corporation, Franklin, MA, USA) during visits 1–4, and collocated with the Discmini. The PDR was run with a triplex cyclone and AirChek pump, calibrated to 1.5 L/minute, as described above.

Twenty-four hour personal and kitchen carbon monoxide concentrations (parts per million, ppm) were measured in visits 1 to 3 for all participants with the Dräger Pac 7000 (Dräger Safety AG & Co., Lubeck, Germany), which was calibrated prior to each field session with 100 ppm carbon monoxide gas. The resolution for the monitors was 2 ppm [36] and was used as the detection limit. Dräger Pac 7000 instruments were set to log at one-minute intervals. For personal measurements, instruments were collocated on the personal monitor bag worn by the participant; for kitchen concentrations, the Dräger Pac 7000 was collocated with the primary kitchen monitors.

### Health endpoints

Table 2 summarizes all health measurements collected in the study, including primary and secondary health endpoints. All health endpoints were based on non-fasting measures. The 3 primary health endpoints were brachial blood pressure (systolic and diastolic), systemic inflammation via CRP, and HbA1c.

**Fig. 3:**
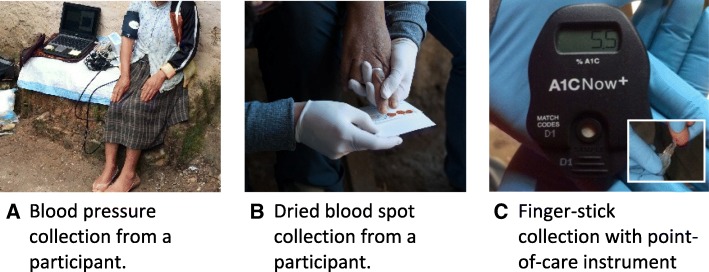
Examples of collection for the primary health endpoints: blood pressure (**a**), dried blood spots C-reactive protein (**b**), and finger-stick sample for glycated hemoglobin (HbA1c) (**c**). Photo credits: Joanna B. Pinneo (**b**)

Brachial systolic and diastolic blood pressure were measured as indicators of CVD risk using the SphygmoCor XCEL Central Blood Pressure Measurement System (AtCor Medical Pty Ltd., West Ryde, Australia), which has also been described in our formative research publication [26]. A brachial artery cuff (23–33 cm or 31–40 cm) was placed on the woman’s right arm while she was in a seated position, with her legs uncrossed and feet resting on the floor (Fig. 3). After 10 min of rest, 3 consecutive measurements were recorded. The woman was asked to refrain from speaking or moving during the readings. The average of the 2nd and 3rd measurements were automatically generated by the device for final systolic and diastolic blood pressure estimates. The SphygmoCor XCEL device received regular calibration and maintenance, as directed by the manufacturer.

To estimate CRP as an indicator of systemic inflammation, dried blood spots were collected via finger-stick from women (Fig. 3). The puncture site on the middle or ring finger on the woman’s non-dominant hand (usually her left hand) was cleaned with 70% isopropanol and allowed to completely dry. A second finger-stick was conducted with the woman’s permission, if the first stick yielded little blood. Following the puncture with the contact-activated safety lancet, the first drop of blood was wiped away with a sterile gauze pad, as this drop may contain an excess of tissue fluids that could cause erroneous results. Up to 5 blood spots were collected onto Whatman protein saver cards, each spot holding 75–80 μl of blood (GE Healthcare Ltd., Forest Farm Industrial Estate, Cardiff, UK). The cards were maintained in a horizontal position during transport back to the field house, dried at room temperature for over 24 h, and stored in a −20 °C freezer in baggies with desiccant and humidity indicator cards in Honduras. Cards were then transported to CSU and stored at −80 °C. Samples will be shipped to the National Health and Environmental Effects Laboratory of the U.S. Environmental Protection Agency (EPA) for analysis, utilizing the V-PLEX Plus Vascular Injury Panel 2 (human) kit for CRP. Women gave consent to store portions of their blood for future measures of possibly important health markers related to smoke exposure.

We measured HbA1c as an indicator of diabetes and metabolic disease risk with the same finger-stick that was used for the blood spots. Approximately 5 μl of blood was used to measure HbA1c, an estimate of average plasma glucose levels for the past 3 months (A1CNow + kit, Bayer Diabetes Care, Sunnyvale, CA, USA) [37].

The secondary health endpoints are briefly described here, with further details in Table 2. Up to 5 dried blood spots were collected as described above for CRP collection, for additional analysis of metabolomics and other biomarkers of systemic injury and inflammation. Central pulse pressure and augmentation index (i.e., an indicator of systemic arterial stiffness) were collected using the same device and procedures as blood pressure (AtCor Medical Pty Ltd., West Ryde, Australia). Waist and hip circumference (measuring tape, cm), weight (scales, kg), height (measuring tape and level against a wall, m) were assessed, and BMI (kg/m^2^) was calculated. Non-fasting blood lipids (mg/dL), including total cholesterol, low-density lipoprotein, high-density lipoprotein, and triglycerides, were measured with the CardioChek kit using 40 μl of blood from a finger-stick (PTS Diagnostics, Indianapolis, IN, USA). To ascertain current respiratory symptoms and symptoms “during cooking activities,” we modified a standardized respiratory symptoms and disease questionnaire developed by the American Thoracic Society [38]. Women self-reported presence or absence of 9 health symptoms at the present moment and also while cooking, including headache, cough, eye irritation, blurry vision, nose irritation, mucous or phlegm, difficulty breathing, wheezing, and throat irritation.

Fractional exhaled nitric oxide (FeNO) was measured to estimate airway inflammation with the NIOX Vero and Aero devices (Circassia Pharmaceuticals Inc., Morrisville, NC, USA). Due to the high costs of the FeNO tests, a subset of 90 women (39%) was randomly selected at the first study visit to conduct the test, and then asked to repeat the test at each subsequent visit. Buccal cells were collected from the same subset of 90 women for visits 1–4 for analysis of telomere length, an indicator of biological aging and potential susceptibility to CVD [39]. Buccal scrapings of left and right sides of the inner cheek were done with 2 child-sized toothbrushes (10–12 scrapes each side, 2 cm head length), preserved in 15 mL of Saccomanno’s buffer, and shipped within 30 days to CSU for storage in a −80 degree Celsius freezer.

Metabolic syndrome, a cluster of cardiometabolic conditions that occur together, will be defined by current international guidelines and modified for non-fasting lipids: waist circumference ≥ 80 cm plus any 2 of the following, triglycerides > 200 mg/dL, high-density lipoprotein < 50 mg/dL, systolic blood pressure ≥ 130 mmHg, diastolic blood pressure ≥ 85 mmHg, and HbA1c > 5.6% [24, 40, 41].

### Additional data

We collected other participant characteristics via in-person questionnaires in tablets using Open Data Kit (ODK Collect 1.4.5, UK) [42]. Other sources of potential air pollution exposure were assessed by asking about smelling smoke from neighboring homes and exhaust from traffic and about practices of trash burning and kerosene use for lighting. Women were asked each visit about their smoking status and exposure to secondhand smoke.

**Fig. 4:**
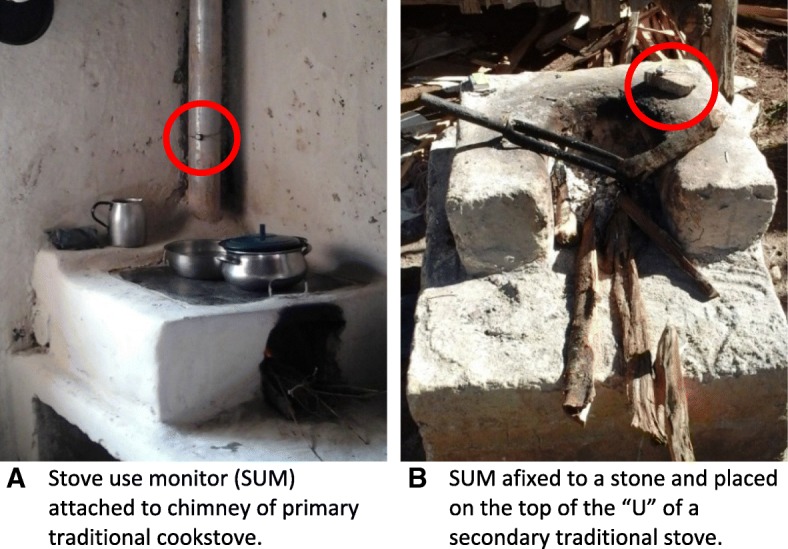
Examples of SUMs on the chimney of a primary traditional cookstove (**a**) and a secondary traditional cookstove (**b**), circled in red

Use of all cookstoves in the household was estimated by logging temperature with electronic cookstove use monitors (SUMs; Thermochron iButtons, Embedded Data Systems, Lawrenceburg, KY, USA). Up to 4 SUMs were deployed per household, depending on the number of cookstoves used. The SUMs could record a range in temperature from 0 to 125 °C and were programmed to log every 5 min for the full 24 h of exposure monitoring. SUMs were placed on chimneys of cookstoves using a harness and silicone buffer to prevent overheating, and if no chimney was present, then SUMs were affixed to a stone and placed near the top of the “U” shape of the chamber (Fig. 4).

Additional stove use and cooking behaviors were measured through self-report in the questionnaires, such as time spent cooking on all stoves and separately for animals, recent use of all stoves, and cooking preferences and opinions. Researchers also completed a separate questionnaire with direct observation to capture maintenance and changes to the *Justa* cookstove.

Household elevation and GPS coordinates were collected at baseline and repeated only if the woman had moved houses during the study. Kitchen characteristics were evaluated by size (height, length, and width), drawn diagrams, housing construction materials (e.g., floor, roof, and wall type), location, and ventilation. Ventilation was measured by number of doors and windows, gaps in the walls (yes/no), eaves between the walls and ceiling (none, less than 30 cm, more than 30 cm), and enclosure status (enclosed, semi-open, open). All kitchen characteristics were collected at baseline and repeated if the kitchen had been changed from the previous visit.

Women’s socioeconomic status and demographic characteristics were estimated through household income sources (agriculture only or agriculture with other sources, such as textiles, selling prepared food, owning a food stand, etc.), household material wealth (number of beds per person for the household and ownership of a bicycle, car, motorcycle, television, radio, refrigerator, cell phone, computer, or sewing machine), household electricity, education (highest level of school completed), age (confirmed by national ID card), and household size. Women also self-reported illnesses, previous doctor diagnoses, medication and vitamin use (confirmed by looking at the bottle or prescription label), 24-h dietary recall and diet diversity from 11 food categories, total years of cooking, physical activity, pregnancy (at screening and each visit), and age of youngest child (to help estimate if she had been unknowingly pregnant in the previous study visit) (Table 2).

Physical activity was estimated as self-reported hours per week (hours per day * days per week) for the following 10 lifestyle activities common to our study population: cut wood, grind corn, wash clothes, milk the cow, work in the field, walk moderately outside the house, cook, clean the house, sit relaxed, and sleep. For each activity, the number of hours per week was multiplied by the corresponding Metabolic equivalent (MET) from the Compendium of Physical Activities (Additional file 3: Table S1) [43]. The values were summed to generate the final score. Descriptions of the activity with its equivalent MET score can be found in Supplemental Materials (Additional file 3: Table S1).

In order to assess dietary intake and dietary diversity, women were asked to report everything they ate and drank in the last 24 h and the number of portions for each item. Our questionnaire listed 20 commonly consumed Honduran food items with photo examples (Additional file 4: Table S2) [44]. A dietary diversity score (DDS) represents the number of food groups consumed over a 24-h period, although the specific number of food groups can vary between populations [45]. We calculated DDS by collapsing the 20 food items into 11 groups: grains (corn, cereals, rice, chips), pulses and nuts (nuts, beans), roots (potatoes), other vegetables, fruits, sweets, eggs, dairy (cheese, milk), meat (beef, chicken, pork, fish), oils, and beverages (coffee, soda, juice). The total DDS was a sum of the dichotomous 11 food groups consumed in the past 24 h, with a minimum score of 1 and maximum of 11. Other studies show DDS to vary by socioeconomic status, food availability, and seasonal changes, especially in low- and middle-income countries (LMIC) [44–46].

### Statistical analysis

We will conduct a descriptive analysis of the exposures, health endpoints, and other measured characteristics (Table 2). Quantitative summaries will include means, standard deviations, ranges, and frequencies as appropriate for the data type. Descriptive statistics will be utilized to assess the similarity of participant characteristics across study arms.

Primary analyses will evaluate the association between assigned cookstove type and the 3 primary health endpoints (blood pressure, CRP, HbA1c) in an intent-to-treat framework. We will use a linear mixed model with a fixed effect for assigned cookstove type (traditional or *Justa*), the independent exposure variable of interest. The model will include a random effect for each participant to account for correlation from repeated measures within person. To account for potential changes in the outcomes over time the model will include visit date using a spline trend function. Letting *Y*_*ij*_ denote the health endpoint measurement for participant *i* at visit *j*, *x*_*ij*_ denote the assigned cookstove type (0 for traditional, 1 for *Justa*) for participant *i* at visit *j*, and *t*_*ij*_ denote the time of visit *j* for participant *i*, this model can be written:

1 $$ {Y}_{ij}=\mu +{a}_i+\beta {x}_{ij}+f\left({t}_{ij}\right)+{\epsilon}_{ij}. $$

In Eq. (1), *β* represents the intervention effect on the outcome measure and is the primary quantity of interest. The parameter *μ* represents the overall average outcome value, *a*_*i*_ represents the random effect for participant *i*, and *ϵ*_*ij*_ is an independent error term. We will use natural cubic splines as the function of time *f* in the model, with the complexity of the adjustment controlled by the number of spline functions.

For the exposure-response analysis, we will use a linear mixed model similar to the model used in the intent-to-treat analysis. In this framework, the personal and kitchen PM_2.5_ measurements will be included as the exposure variable *x*_*ij*_ in Eq. (1). As the exposure-response approach will not utilize the study’s randomization to control for confounding, potential confounders identified a priori will be included in Eq. (1) as additional fixed effect terms. Potential confounders include participant age, indicators of socioeconomic status (e.g., household possessions, participant education level, beds per person in each household), physical activity levels, BMI, and dietary intake. We will also conduct a “per protocol” analysis using self-reported actual cookstove use, rather than assigned cookstove.

We will evaluate effect modification by adding the following variables as interaction terms with the stove assignment or exposure measurement variable in separate models: age (< 40 or ≥ 40 years), blood pressure (normal systolic < 120 and diastolic < 80 vs. borderline high/high systolic ≥ 120 or diastolic ≥ 80), diabetes status (normal hbA1c < 5.7% vs. pre-diabetic/diabetic ≥ 5.7%), BMI (normal < 25 or overweight/obese ≥ 25), waist circumference (< 80 cm vs. ≥80 cm), and metabolic syndrome (presence vs. absence).

Sensitivity analyses will be conducted to assess the impact of different forms of the adjustment for time in Eq. (1) by varying the number of spline functions and considering alternative representations such as indicators of season or visit number. We will also conduct sensitivity analyses that exclude women who were taking medications that could influence inflammation in the body, such as hypertension or vitamins, or who were ill at the time of the health measurements. Additional sensitivity analyses will exclude air pollution samples that came from pumps that ran for less than 24 h, and flow rates that strayed outside the 10% pre- and post-sampling range.

The sample size of 230 was selected based on feasibility and cost considerations and is well-powered for the primary analyses. We estimated power by conducting a simulation that generates data according to the study design and Eq. (1). For the blood pressure model: assuming a standard deviation of 12 mmHg for systolic blood pressure [26], a correlation of 0.76 between repeated measures from the same individual [47], and a 10% loss to follow-up, we estimate there will be 80% power to detect a difference of 1.81 mmHg. This difference is smaller than that observed in the only cookstove randomized controlled trial conducted among non-pregnant women in Guatemala [7]. The power remained constant adding linear trends of 3 mmHg/year and seasonal trends of amplitude 3 mmHg to the simulated blood pressure measurements. For the CRP model: assuming a standard deviation of 1.1 mg/dL [48], a correlation of 0.66 [49] between repeated measures from the same individual, and a 10% loss to follow-up, we estimate there will be 80% power to detect a difference of 0.2 mg/dL. Although different cooking fuels and sources of indoor pollution were investigated, this difference in CRP is similar to that observed in a random crossover design evaluating the impact of indoor air filtration among homemakers in Taiwan [50].

For missing data, we will record the number, timing, pattern, and reason. Missing data may occur due to subject-initiated drop out, missing visits, instrument failure, and data entry error. We will evaluate whether the missing data patterns are differential between study arms, or by study visit. For all analyses, we will assume missing data to be missing at random a priori.

Data will be analyzed using SAS® software version 9.4 (SAS Institute, Inc., Cary, NC, USA) and R Statistical Software (The R Project for Statistical Computing).

### Data management and confidentiality

All electronic data will be kept on a password-protected, secured drive at CSU, accessible only to researchers on this project. Hard copies of exposure recording forms are kept in a locked filing cabinet at CSU. All data files use a unique household ID for participants, with the link between IDs and names only available to the researchers in a protected file on the secured drive. Data files sent to/from the EPA lab with biomarker results will use de-identified information and the unique household IDs.

### Ethics approval and data monitoring

This study was approved by the CSU Institutional Review Board (#12-3870H). Due to low literacy rates in the study area, verbal informed consent was obtained from all participants prior to enrollment and at each study visit. Women were reminded at each visit that their participation was completely voluntary and that all personal data would be kept confidential. The CSU Institutional Review Board reviewed and approved any protocol modifications and study amendments, as well as any reported adverse events.

The Data Monitoring Committee (DMC) at CSU conducted ongoing reviews of the trial over the course of the 3-year study. Five reports were submitted to the DMC to provide reviewers information on current recruitment and attrition, preliminary results for primary health endpoints by study arm, and reasons for temporary and permanent exclusions of participants. In these reports, we also reported potential adverse events and preliminary results based on descriptive summaries of participant sociodemographic and health characteristics. Any concerns raised by the DMC were addressed in the study and action steps were described in the following report to demonstrate the research team’s response to any issues. No interim analyses were planned due to the nature of the intervention and the timeline of the study.

### Dissemination of results

Results from this research are planned to be disseminated to local stakeholders in Honduras, including officials of the Ministry of Health, local mayors near La Esperanza, community leaders, and participants and their family members. Results will also be disseminated through professional conferences and peer-reviewed publications. Trial results will also be posted and updated on the U.S. National Library of Medicine clinicaltrials.gov website, when available.

## Discussion

Household air pollution from biomass-burning stoves is a major public health threat that has yet to be fully characterized in terms of its global burden for morbidity and mortality. Reducing household air pollution is possible if households transition away from open-fire and inefficient traditional cookstoves. Clean cooking and use of solid-fuel cookstoves designed with engineered combustion chambers and chimneys can reduce emissions, yet health impacts from cookstove interventions are largely inconclusive [16]. Poor adoption and sustained use are often key challenges for long-term success of cookstove interventions. Few randomized controlled trials have been conducted to help further our understanding of effective cookstove solutions with measurable health benefits.

To address these gaps, this study seeks to integrate a community-engaged approach into a cookstove intervention. Careful selection of a culturally accepted and preferred cookstove with guidance from community members is crucial for participant acceptance, maintenance, and proper use. Through our intervention using the Honduran-made and community-vetted *Justa* cookstove, we will evaluate its effects of reducing household air pollution and improving indicators of cardiometabolic- and respiratory-related health endpoints, while understanding barriers to new cookstove adoption.

We utilized a stepped-wedge design, relatively novel and increasing in popularity since all participants receive the intervention, which may address stakeholder concerns if there is a perceived ethical dilemma in withholding the intervention from some participants [31, 51]. Compared to the more conventional “parallel” cluster randomized trial where assigned intervention or control arm does not change, the stepped-wedge design allows pre- and post-intervention observations for both study arms, since both arms receive the intervention at different time points.

The primary health endpoints of indicators of CVD (blood pressure and CRP) and diabetes risk (HbA1c) were selected in response to the substantial global burden of premature mortality and morbidity from cardiometabolic diseases [6]. The growing evidence of an association between ambient and household air pollution with cardiometabolic disease risk [7–11, 13, 52] makes air pollution a modifiable risk factor with the potential for prevention on a global scale. Furthermore, the use of dried blood spots collected with a finger-stick by trained non-medical staff offers a “field-friendly” and less invasive procedure for collecting blood samples, with easier transportation and storage requirements compared to venous-drawn blood [53]. CRP from blood spots was highly correlated with paired serum from venous-drawn blood (Pearson R = 0.96) [53], supporting dried blood spots as a feasible approach to evaluate household air pollution’s impact on systemic inflammation in field settings [54, 55].

We used SUMs and accelerometers to capture objective information on stove use and compliance with wearing personal monitors, respectively. The SUMs will be used to quantify cooking events and time of stove use, which can be compared with women’s self-reported use. This information will also provide insights into continued use of traditional stoves and other stove types. The accelerometers, collocated with the other personal exposure instruments, will offer insights into the women’s compliance with wearing the personal equipment. The monitors recorded 3-axis movement data and can be used to track how often the bag/necklace was removed and left sitting, rather than being worn throughout the day, apart from sleeping.

### Limitations

Several limitations are important to note. While traditional stoves in women’s homes were destroyed before *Justa* construction, secondary traditional stoves could be easily remade. We anticipate that exclusive use of *Justa* cookstoves for the full study period would be difficult to achieve, as families in this region often relied on secondary stoves to cook for holidays or large family gatherings, boil corn in large pots for tortillas, and roast coffee beans. Women were not excluded from the study if they continued using traditional cookstoves after the *Justa* intervention. Near-complete displacement of traditional stoves is considered necessary to see health benefits [23], and our PM_2.5_ levels from *Justa* users will likely remain above the WHO guideline of 25 μg/m^3^ for a 24-h mean in many of the households [4].

A major limitation of this study was that blinding for participants and field team members was not possible given the type of intervention. An additional limitation to this study is our lack of ambient air pollution data that might affect women’s longer-term exposures, such as emissions from burning agricultural fields, neighbors’ biomass combustion, diesel traffic fumes, and trash burning. We attempted to capture these additional sources of exposure in the questionnaires at each visit to control for these co-occurring exposures during analysis.

## Conclusion

While other sources of household energy, such as liquefied petroleum gas and electricity, have lower emissions and potentially greater health impacts than a solid-fuel engineered stove, these fuel options are still not affordable or easily accessible for our Honduran study population. Given the current needs and availability of cookstove designs in this region, the *Justa* may be the most realistic option to reduce household air pollution. We will offer a complete assessment of the links between stove use, exposure measures, and health outcomes. Our larger goal will be to use findings from this trial to better understand if cardiometabolic disease risk can be meaningfully reduced following a household-level cookstove intervention, and to inform the gap in knowledge in the global burden of disease from household air pollution, especially in LMICs.

## Additional files

Additional file 1: **Figure S1.** *Justa* cookstove training poster, English version. Posters were printed on water-resistant laminate material, hung near the *Justa* cookstove, and reviewed during in-person training and subsequent study visits. (PDF 564 kb)

Additional file 2: **Figure S2.** Participant health feedback form, English version. Participant’s results were circled with the corresponding status, and results were explained. As mentioned in the form, participants were encouraged to visit a community health center or physician if they had any further questions or concerns. (PDF 525 kb)

Additional file 3: **Table S1.** Physical activities with descriptions and MET score from the 2011 Compendium of Physical Activity. (DOCX 16 kb)

Additional file 4: **Table S2.** Twenty commonly consumed Honduran food items included in the dietary recall section of the health questionnaire. (DOCX 15 kb)

## Data Availability

Specialized standard operating protocols and questionnaires/surveys will be available upon request from the corresponding author. All peer-reviewed publications from this trial will be published as open access articles.

## Abbreviations

AHDESA: Asociación Hondureña para el Desarrollo
BMI: Body mass index
CI: Confidence interval
CRP: C-reactive protein
CSU: Colorado State University
CVD: Cardiovascular disease
DDS: Dietary diversity score
DMC: Data Monitoring Committee
EPA: Environmental Protection Agency (U.S.)
FeNO: Fractional exhaled nitric oxide
FOCAEP: Fondo Centroamericano para el Acceso a la Energía y Reducción de la Pobreza
HbA1c: Glycated hemoglobin (hemoglobin A1c)
LMICs: Low- and middle-income countries
LOD: Limit of detection
MET: Metabolic equivalent
PM: Particulate matter
PM_2.5_: Fine particles < 2.5 μm in aerodynamic diameter
PNC: Particle number concentration
PPM: Parts per million
SUM: Stove use monitors (electronic temperature logger)
TWP: Trees, Water & People
UPAS: Ultrasonic Personal Aerosol Sampler
WHO: World Health Organization
