# Supplementary material for: Study protocol for a stepped-wedge randomized cookstove intervention in rural Honduras: household air pollution and cardiometabolic health
Source: BMC Public Health. 2019 Jul 8;19:903. doi: 10.1186/s12889-019-7214-2 (PMC6615088; doi:10.1186/s12889-019-7214-2)
Supplement: Supplementary file 1 — Figure S1. Justa cookstove training poster, English version. Posters were printed on water-resistant laminate material, hung near the Justa cookstove, and reviewed during in-person training and subsequent study visits. (PDF 564 kb) [file 12889_2019_7214_MOESM1_ESM.pdf]

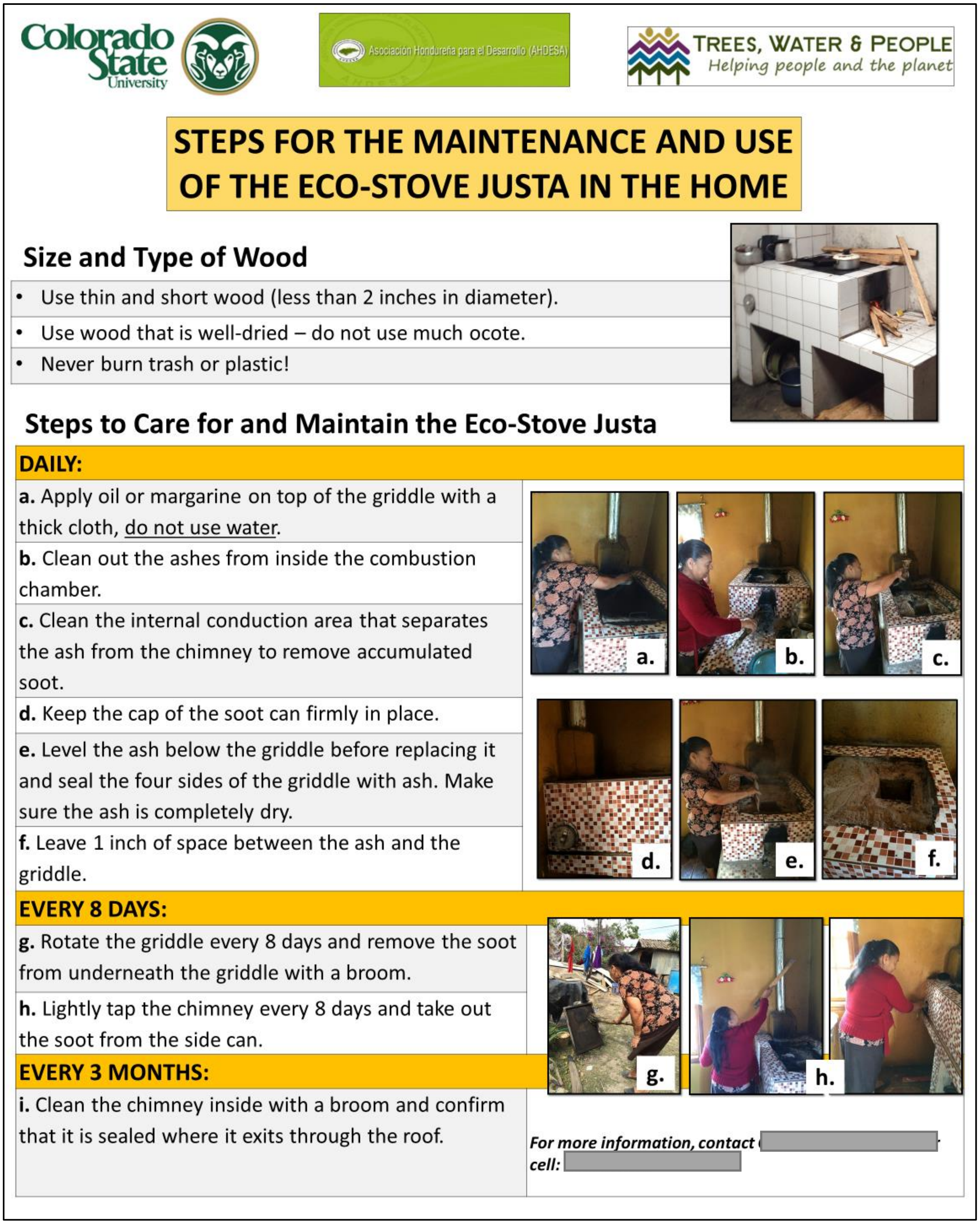

Figure S1. *Justa* cookstove training poster, English version. Posters were printed on water-resistant laminate material, hung near the *Justa* cookstove, and reviewed during in-person training and subsequent study visits.
