# Supplementary material for: Study protocol for a stepped-wedge randomized cookstove intervention in rural Honduras: household air pollution and cardiometabolic health
Source: BMC Public Health. 2019 Jul 8;19:903. doi: 10.1186/s12889-019-7214-2 (PMC6615088; doi:10.1186/s12889-019-7214-2)
Supplement: Supplementary file 2 — Figure S2. Participant health feedback form, English version. Participant’s results were circled with the corresponding status, and results were explained. As mentioned in the form, participants were encouraged to visit a community health center or physician if they had any further questions or concerns. (PDF 525 kb) [file 12889_2019_7214_MOESM2_ESM.pdf]

Participant Health Measures

Colorado State University | Honduras Cookstove Project

Date: ______________ **Name:** _________________________ **Age:** ______ years

General

My weight is: ________ pounds

My height is: ________ inches

My body mass index (BMI) is: ______

My blood pressure is: ______ / ______

What do these numbers mean?

Adult BMI	Status
Below 18.5	Underweight
18.5 – 24.9	Normal
25.0 – 29.9	Overweight
30.0 and higher	Obese

Blood Pressure (mmHg)	Status
Less than 120 / 80	Normal
120-139 / 80-89	Prehypertension
140 / 90 and higher	High

Diabetes

My hemoglobin A1c is (measure of blood sugar): ______ %

What do these numbers mean?

Hemoglobin A1c (%)	Status
4 - 5.6	Normal
5.7 - 6.4	Prediabetes
6.5 or higher	Diabetes

Blood Lipids (non-fasting)

My total cholesterol level is: _______ mg/dL

My triglyceride level is: __________ mg/dL

What do these numbers mean?

Total cholesterol (mg/dL)	Status
200 or less	Normal
201 – 239	Borderline high
240 and higher	High

Triglyceride level (mg/dL)	Status
Less than 150	Normal
150 – 199	Borderline high
200 or higher	Very high

NOTE: *We are not doctors and cannot diagnose or treat health problems. These results are general measures of your health; they are not meant to diagnose a disease. If you have any questions or concerns with your numbers, please see your community health center or physician.*

Figure S2. Participant health feedback form, English version. Participant's results were circled with the corresponding status, and results were explained. As mentioned in the form, participants were encouraged to visit a community health center or physician if they had any further questions or concerns.
