# Supplementary material for: Study protocol for a stepped-wedge randomized cookstove intervention in rural Honduras: household air pollution and cardiometabolic health
Source: BMC Public Health. 2019 Jul 8;19:903. doi: 10.1186/s12889-019-7214-2 (PMC6615088; doi:10.1186/s12889-019-7214-2)
Supplement: Supplementary file 3 — Table S1. Physical activities with descriptions and MET score from the 2011 Compendium of Physical Activity. (DOCX 16 kb) [file 12889_2019_7214_MOESM3_ESM.docx]

Table S1. Physical activities with descriptions and MET score from the 2011 Compendium of Physical Activity.[1]

| **Activity** | **Code** | **Description** | **MET** |
| --- | --- | --- | --- |
| Cutting wood | 08010 | carrying, loading or stacking wood, loading/unloading or carrying lumber | 5.5 |
| Grinding corn | 05035 | kitchen activity, general, (e.g., cooking, washing dishes, cleaning up), moderate effort | 3.3 |
| Washing clothes | 05092 | laundry, hanging wash, washing clothes by hand, moderate effort | 4 |
| Milking cow | 11210 | farming, milking by hand, cleaning pails, moderate effort | 3.5 |
| Working in the field | 11146 | farming, moderate effort (e.g., feeding animals, chasing cattle by walking and/or horseback, spreading manure, harvesting crops) | 4.8 |
| Walking moderate | 05165 | walking, moderate effort tasks, non-cleaning (readying to leave, shut/lock doors, close windows, etc.) | 3.5 |
| Cooking | 05035 | kitchen activity, general, (e.g., cooking, washing dishes, cleaning up), moderate effort | 3.3 |
| Cleaning | 05010 | cleaning, sweeping carpet or floors, general | 3.3 |
| Sitting | 05080 | knitting, sewing, light effort, wrapping presents, sitting | 1.3 |
| Sleeping | 07030 | sleeping | 0.95 |

REFERENCES

1. Ainsworth, B.E., et al., *2011 Compendium of Physical Activities: a second update of codes and MET values.* Med Sci Sports Exerc, 2011. **43**(8): p. 1575-81.
