# Supplementary material for: Study protocol for a stepped-wedge randomized cookstove intervention in rural Honduras: household air pollution and cardiometabolic health
Source: BMC Public Health. 2019 Jul 8;19:903. doi: 10.1186/s12889-019-7214-2 (PMC6615088; doi:10.1186/s12889-019-7214-2)
Supplement: Supplementary file 4 — Table S2. Twenty commonly consumed Honduran food items included in the dietary recall section of the health questionnaire. (DOCX 15 kb) [file 12889_2019_7214_MOESM4_ESM.docx]

Table S2. Twenty commonly consumed Honduran food items included in the dietary recall section of the health questionnaire.

| **Number** | **Food item** | **Examples** |
| --- | --- | --- |
| 1 | corn | Tortillas, cob, atole |
| 2 | grains | Cereal, oatmeal, other grains (bread, spaghetti) |
| 3 | rice | Rice |
| 4 | chips | Chips |
| 5 | beans | Beans, lentils, peas |
| 6 | nuts | Peanuts, almonds, other nuts, seeds |
| 7 | starches | Potatoes, yams, cassava |
| 8 | vegetables | Carrot, broccoli, greens, cauliflower |
| 9 | fruit | Banana, orange, papaya, pineapple, melon, mango |
| 10 | sweets | Candies, chocolate, cookies, cake, sweet bread, dessert |
| 11 | eggs | Eggs |
| 12 | cheese | Cheese, cuajada |
| 13 | milk | Milk, cream, butter |
| 14 | chicken | Chicken |
| 15 | beef | Steak, hamburger, carne asada |
| 16 | pork | Pork |
| 17 | fish | Fish and seafood |
| 18 | fat | Oils and fat |
| 19 | coffee | Coffee |
| 20 | Sweet drinks | Soda, juice |
